# Cost-utility analysis of increasing uptake of universal seasonal quadrivalent influenza vaccine (QIV) in children aged 6 months and older in Germany

**DOI:** 10.1080/21645515.2022.2058304

**Published:** 2022-04-29

**Authors:** Daniel Molnar, Anastassia Anastassopoulou, Barbara Poulsen Nautrup, Ruprecht Schmidt-Ott, Martin Eichner, Markus Schwehm, Gael Dos Santos, Bernhard Ultsch, Rafik Bekkat-Berkani, Alfred von Krempelhuber, Ilse Van Vlaenderen, Laure-Anne Van Bellinghen

**Affiliations:** a GSK, Wavre, Belgium; b Health Outcomes and New Products, GSK, Munich, Germany; c EAH-Consulting, Aachen, Germany; d Global Medical Affairs, GSK, Wavre, Belgium; e Epimos GmbH, Bischofsheim, Germany; f University of Tübingen, Tübingen, Germany; g ExploSYS GmbH, Leinfelden-Echterdingen, Germany; h GSK, Munich, Germany; i Global Medical Affairs Vaccines, GSK, Rockville, MD, USA; j CHESS in Health, Bonheiden, Belgium

**Keywords:** Children, economic model, Germany, quadrivalent influenza vaccine, seasonal influenza, uptake

## Abstract

Seasonal influenza causes many cases and related deaths in Europe annually, despite ongoing vaccination programs for older adults and people at high-risk of complications. Children have the highest risk of infection and play a key role in disease transmission. Our cost-utility analysis, based on a dynamic transmission model, estimated the impact of increasing the current vaccination coverage with inactivated quadrivalent influenza vaccine in Germany to all (healthy and high-risk) children under 5 years of age (40% uptake), or under 18 years (40% uptake), or only high-risk children under 18 years (90% uptake). Eight influenza complications were modeled, hospitalization and death rates were based on age and risk status. All three vaccination strategies provided more health benefits than the existing vaccination situation, reducing influenza cases, complications, hospitalizations and deaths across the entire population. The strategy targeting all children under 5 years was highly cost-effective (€6/quality-adjusted life-year gained, payer perspective). The other strategies were cost saving from the payer and societal perspectives. The vaccination strategy targeting all children under 18 years was estimated to provide the most health benefits (preventing on average 1.66 million cases, 179,000 complications, 14,000 hospitalizations and 3,600 deaths due to influenza annually) and the most cost savings (annually €20.5 million and €731.3 million from payer and societal perspectives, respectively). Our analysis provides policy decision-makers with evidence supporting strategies to expand childhood influenza vaccination, to directly protect children, and indirectly all other unvaccinated age groups, in order to reduce the humanistic and economic burden on healthcare systems and society.

## Plain Language Summary

**What is the context?**

- Every winter, millions of people in Europe become ill due to influenza (flu), and some need to be hospitalized for complications that can sometimes lead to death.
- While mainly older adults and people with chronic illness are at higher risk of complications from influenza, children have the highest risk of infection and of transmitting the disease.
- Current vaccination policies in Europe, including Germany, target older adults and high-risk populations (pregnant women, children and other age groups with chronic diseases).

**What is new?**

- This analysis simulates the effects of expanding current German vaccination programs in high-risk children to include healthy children, and of increasing vaccination coverage rates, for direct protection against infection, and to reduce the disease transmission in the rest of the population.
- We modeled three vaccination strategies:
  - vaccinating 40% of all (healthy and high- risk) children under 5 years old;
  - vaccinating 40% of all (healthy and high-risk) children under 18 years old;
  - vaccinating 90% of high-risk children under 18 years old.

**What is the impact?**

- All three strategies resulted in health gains, as more influenza cases, complications and deaths were prevented in all age groups of the population compared to the current situation.
- The strategies targeting both healthy and high-risk children provided the greatest health benefits. In particular, a vaccination policy targeting all children under 18 years old was predicted to provide the most health benefits as well as the highest cost savings: the increased costs of vaccination were more than offset by the savings in disease management costs as a result of having fewer influenza patients.
- Vaccinating healthy children against influenza is expected to significantly reduce the disease burden in the total population while saving costs, due to reduced transmission of the disease.

## Introduction

Every winter, seasonal influenza epidemics due to influenza A and B viruses affect large numbers of people (4–50 million symptomatic cases) in the European Union/European Economic Area (EU/EEA), with the associated risk of complications, hospitalization and death (15,000–70,000 influenza-associated deaths).^1^ The risk of complications and severe disease is highest in adults over 65 years of age and people with chronic conditions, while young children are at higher risk of infection and transmission, as they have no or low levels of prior immunity through previous exposure.^1^ Although the consequences of infection are generally less severe in children, the large number of mild to moderate cases also place a significant burden on health services and on society from lost productivity.^1^

In Germany, during the 2018/2019 winter season, influenza was responsible for an estimated 3.8 million (95% confidence interval [CI] 3.0–4.6 million) medically-attended acute respiratory cases, 18,000 (95% CI 16,000–20,000) hospitalizations and around 2.3 million (95% CI 2.1–2.5 million) cases with lost productivity.^2^ As in previous seasons, a large proportion of hospitalized cases were aged under 5 years of age.^2,3^

The economic burden in Germany was estimated in a matched case–control study using 2012–2014 data from a large sickness fund.^3^ Average annual direct costs were estimated to be around €78 million (between €53 million in 2014 and €121 million in 2012), with highest costs in the youngest and oldest age groups. The highest risk of hospitalization was in infants and young children. Influenza cases, especially children under 6 years and adults over 60 years, were significantly more likely to have complications e.g., acute otitis media (AOM) and community-acquired pneumonia (CAP), which contributed to higher inpatient costs. Around 33% of influenza cases took, on average, 6.7 days of sick leave.^3^

Children play an important role in the transmission of influenza to adults and older adults, as they experience a longer duration of viral shedding.^4^ In the United States, an analysis of different age groups’ influence on transmission during influenza epidemics from 2009 to 2014 found that children aged 5–17 years were the most important drivers of transmission.^5^ Modeling studies (including in Germany in 2014^6^) have estimated that routine childhood influenza vaccination campaigns could considerably reduce the disease burden in children as well as in older age groups by reducing community transmission (i.e., providing herd protection),^6,7^ with greater success related to higher vaccine uptake rates.^8–10^ Therefore, universal influenza mass vaccination in children offers an alternative strategy to reducing the high burden of seasonal influenza in the entire German population.

The World Health Organization (WHO) recommends seasonal influenza vaccination, as the most effective way to prevent disease, for children aged 6 months to 5 years, as well as for other age and risk groups.^11^ Several safe and effective vaccines are available and recommended from the age of 6 months, with quadrivalent influenza vaccines (QIVs) providing broader protection than trivalent influenza vaccines (TIVs), as they include protection against an additional influenza B virus lineage.^12,13^ In the EU/EEA, influenza vaccination is typically reimbursed for adults from the age of 60 years and other risk groups (e.g., pregnant women and people with chronic conditions), but few countries include seasonal influenza vaccination for healthy children in their National Immunization Program.^14^ In Germany, it is recommended only for children at risk of complications.^15^

An economic model was developed, based on the epidemiologic outputs of the published 4Flu dynamic transmission model,^16–18^ in order to evaluate the public health and economic impact of broader vaccination strategies in Germany, including healthy children and adolescents. A cost-utility analysis was conducted, increasing QIV uptake in several pediatric populations i.e., in all children under 5 or under 18 years, or only in high-risk children under 18 years.

## Materials and methods

### Population and setting

An economic model (e4Flu) was developed, using the predicted number of influenza cases derived from the 4Flu individual-based dynamic transmission model,^17^ to evaluate the impact of universal pediatric vaccination strategies on influenza burden in the entire German population, versus the current influenza vaccination situation (GSK study identifier: HO-17-18828). The current situation (base case) assumed pediatric QIV was used with an uptake of 4.1%,^19^ in addition to 13.6–22.2%^20^ uptake for adults under 60 years old and 33.2%^21^ for adults aged 60 years old and above. All three strategies maintained the same vaccination uptake in adults and older adults, derived from the literature, but varied pediatric QIV uptake rates as follows: Strategy A) 40% uptake in children aged 6 months to 4 years; Strategy B) 40% uptake in children aged 6 months to 17 years; and Strategy C) 90% uptake in high-risk children aged 6 months to 17 years.

### Model overview, inputs and assumptions

**Figure 1.**
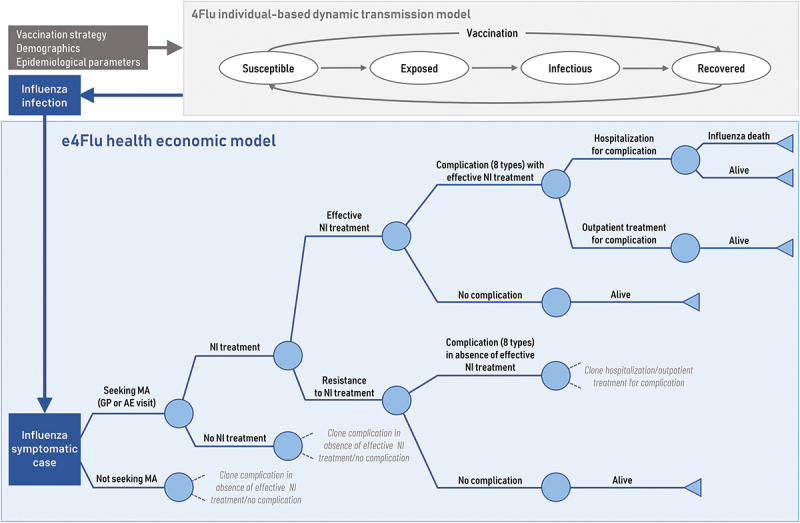
Model structure with epidemiologic (4Flu) and economic (e4Flu) pathways. *Source*: Adapted from Dolk et al.^31^ Abbreviations: AE, accident & emergency; GP, general practitioner; MA, medical advice; NI, neuraminidase inhibitor.

The economic e4Flu model is a decision tree model (Figure 1) for symptomatic influenza cases, which includes probabilities (age-specific and by “high-risk” or “healthy” status^22^ and associated costs and (dis)utilities of using healthcare resources (i.e., general practitioner [GP] or Accident & Emergency [AE] visits, Neuraminidase Inhibitor [NI] treatment, hospitalization, outpatient visits), as well as the risk of developing mutually-exclusive complications (i.e. upper respiratory tract infection [URTI], AOM, bronchitis, pneumonia, gastrointestinal bleeding, cardiac, renal or central nervous system complications), and of influenza-associated mortality, conservatively assumed only for influenza cases with a hospitalized complication. See Supplementary Tables S1, S2 for model probabilities, (dis)utilities, and cost inputs.

Each health state is associated with an age-specific direct and indirect cost (due to lost productivity and premature mortality), and disutility (baseline utilities differ by age and risk group), derived from the literature (Supplementary Table S1). For example, reimbursed hospital costs for the more common complications included bronchitis (€3,023), pneumonia (€4,228), URTI (€2,243) and AOM (€1,475) among others (Supplementary Table S2). Effective NI treatment, prescribed during a GP or AE visit, was assumed to reduce the duration of symptomatic influenza from 7.5 days^23^ to 6.5 days^24^ but did not affect the risk of complications. Age-specific but risk group-independent disutilities were assumed for all hospitalized complications (i.e., .54 for <18 years, .60 for 18–49 years, .58 for 50–64 years, and .56 for ≥65 years,^23^) and all outpatient complications (i.e., .41 for <18 years, .47 for 18–49 years, .36 for 50–64 years, and .32 for ≥65 years^23^), with duration varying by type of complication (e.g., from 3.22 days for AOM to 9.62 days for renal complications,^25,26^ see Supplementary Table S2). Outpatient visits for complications were conservatively assumed to have the same disutility as an uncomplicated case.

Influenza infections (66.9% of which were assumed to be symptomatic cases^27^) over a 20-year timeframe (from 1^st^ September 2017 to 2036) for the base case and each strategy were predicted from the published 4Flu transmission model,^16,17,28^ an individual-based stochastic tool that simulates the independent spread of 4 influenza viruses (i.e., influenza A virus subtypes A/H1N1 and A/H3N2, and influenza B virus lineages B/Yamagata and B/Victoria) in the German population with dynamically changing demography^29^ and contact patterns (based on the German POLYMOD study).^30^ The 4Flu model uses a SEIRS structure i.e., individuals move between health states: susceptible, exposed (latent), infectious, recovered, susceptible (Figure 1, adapted from Dolk et al.^31^). The 4Flu model used German age-specific demographics, vaccination strategy parameters and influenza epidemiology parameters as inputs. As described elsewhere,^18^ major antigenic drift events were assumed to occur in random years (on average every 3.5 years for A(H3N2) and every 7 years for the other influenza variants). Against newly circulating drift variants, only 60% of the previously immune population were assumed to be immune. In 40% of the drift years, the vaccine was assumed not to be well matched against the newly circulating variant, which was modeled by a 40% reduction in vaccine efficacy.^18^ A 20-year run-in period was applied (1997–2016) using TIV, to ensure that the starting model population provided a realistic age-dependent immunity pattern for the German population. During the 20-year analysis period starting on 1^st^ September 2017, the base case and all evaluated strategies used QIV, with only pediatric vaccination uptake rates differing between each strategy.

### Vaccine efficacy and uptake

Vaccine efficacy data were obtained from available literature (Table 1). In line with other studies, no adverse events were considered in the model since adverse events related to influenza vaccination are usually mild and self-limiting.^32,33^ Children under 9 years of age receiving their seasonal influenza vaccination for the first time (naïve recipients) receive two doses separated by at least 4 weeks, as per official guidance,^34^ however, the model assumed the same efficacy as for a single dose in non-naïve individuals.

**Table 1.** Vaccination uptake rates (base case) and efficacy.

Age group	Reported uptake (%)	Calculated uptake (%)		Vaccine efficacy (%)
		Healthy	High-risk ^a^	
6 months–2 years	4.1^19,b^	3.87^18,b^	7.74^18,b^	49.8^35^
3–8 years	4.1^19,b^	3.87^18,b^	7.74^18,b^	55.4^36^
9–15 years	4.1^19,b^	3.87^18,b^	7.74^18,b^	69.0^37^
16–17 years	4.1^19,b^	3.59^18^	7.18^18^	63.0^38,b^
18–39 years	13.6^20^	11.91^18^	23.82^18^	63.0^38,b^
40–59 years	22.2^20^	19.44^18^	38.88^18^	63.0^38,b^
60–64 years	33.2^21,b^	33.2^18,b^	33.2^18,b^	63.0^38,b^
65+ years	33.2^21,b^	33.2^18,b^	33.2^18,b^	58.0^39^

^a^Proportion high-risk: 1–15 years (6.0%); 16–59 years (14.2%); >59 years (47.1%)^22^; ^b^reported age group data (e.g., for age group 6 months to 17 years) applied to model age groups.

Age-specific vaccination uptake rates reported in the literature were used to estimate base case uptake rates for healthy and high-risk groups in the model (Table 1), assuming that uptake is twice as high in high-risk children and high-risk adults under 60 years (based on Advisory Board input, March 2017, Frankfurt). The model assumed it would take 5 years to reach the maximum vaccination uptake. The 40% uptake assumption for children as applied in Strategies A and B was based on a survey by the Robert Koch Institute of parents in Germany, showing that over half would vaccinate their children against seasonal influenza if the vaccine was recommended.^40,41^

The cost of QIV vaccination was estimated at €13.11 (based on the average of official QIV vaccine prices weighted by their market share) and an administration cost of €7.70 (based on the reimbursed cost of administration by a physician). See Supplementary Table S1 for more details.

### Analysis

The model calculated direct and indirect costs and quality-adjusted life-year (QALY) loss associated with symptomatic influenza cases, to estimate the incremental cost per QALY gained with each strategy versus the base case, from a payer and societal perspective. As there is no official willingness-to-pay threshold in Germany, the commonly used threshold of €50,000 per QALY gained was assumed (i.e., around 1x gross domestic product per capita, using the WHO recommended threshold for a highly cost-effective intervention), as in other analyses.^31,41–43^

The e4Flu model used a starting cohort of 100,000 individuals for 2017, with age- and at-risk distribution representative of the German population. With around 82.6 million inhabitants in Germany in 2017, a factor of 826 was applied when extrapolating the model results to the entire population.^44^ As the analysis covers a period of 20 years, reported annual results were based on an average of these 20-year findings.

The payer perspective included reimbursable medical costs and the non-medical costs of the German child sick pay benefit (“*Kinderkrankengeld*”), which covers 90% of parental net earnings for absenteeism to care for a sick child up to the age of 12 years.^45^ In addition to these, the societal perspective included non-reimbursable medical costs (out-of-pocket costs of over-the-counter medication), non-reimbursable non-medical costs (transportation costs) and indirect costs of lost productivity (in adults aged 18–64 years), as well as parental absenteeism for a sick child that is not covered by the child sick pay benefit. Indirect costs due to premature mortality were estimated using the friction cost approach.^46^

Costs and health outcomes were discounted at 3% per annum following German health economic guidelines.^46^ All costs were expressed as 2017 Euro (€) values, where necessary, costs were inflated using the consumer price index.

### Scenario analyses

Due to the 20-year time horizon, and according to German guidelines, Scenario analysis 1 assessed the impact of differentially discounting health outcomes at 1% while maintaining the discount rate for costs at 3%.^46^

Baseline age-specific utilities were derived from Garcia et al.^23^ for Spain, as they allowed different utility values to be used for healthy and high-risk populations. In Scenario analysis 2, German utility values were used instead, with no distinction by risk status.^47^

### Uncertainty analyses

The impact of parameter uncertainty was assessed using probabilistic sensitivity analysis (PSA), with 1,000 iterations. Parameter values were randomly drawn from their associated distributions (e.g., lognormal distribution for vaccine efficacy, duration of naturally acquired immunity, average circulation time per drift variant, and beta distribution for infection probability per contact and cross-protection for drift mismatch) in the 4Flu model, resulting in 1,000 sets of parameters. As 4Flu is a stochastic model, each set was used to run 100 simulations, and average results were coupled to independent random samples from the probability distributions of economic e4Flu model parameters (e.g., gamma distribution for costs or disutilities relating to influenza, beta distribution for probabilities). The results of the PSA are presented in a cost-effectiveness plane.

## Results

Increasing QIV uptake in all three pediatric populations reduced the number of influenza cases in the vaccinated age group,^18^ as well as in all age groups across the German population over a period of 20 years. As a consequence, the model predicted fewer complications and deaths, and a resulting gain in health outcomes. Despite increased vaccination costs for each strategy compared to the base case, there was a substantial decrease in direct and indirect, reimbursable and non-reimbursable influenza costs due to the reduction in cases. From the payer perspective, this led to overall cost savings for Strategies B and C (increasing uptake in all children under 18 years or only in high-risk children), and to a cost-effective result of €6 per QALY gained for Strategy A (increasing uptake in all children under 5 years). From a societal perspective, all three strategies led to cost-savings compared with the base case. The most important health benefits and largest cost savings were seen with strategies increasing uptake in all children below the age of 18 years (Table 2).

**Table 2.** Influenza burden, QALYs gained and incremental costs (3% discounted) over 20 years (per 100,000 population), and incremental costs per QALY gained by strategy versus the base case.

	**All children**			**High-risk children**	
	**Base case for A and B**^a^	**Strategy A** **(40% uptake, 6m–4y)**	**Strategy B** **(40% uptake, 6m–17y)**	**Base case for C^a^**	**Strategy C** **(90% uptake, HR 6m–17y)**
Influenza cases	177,154	168,989	137,051	175,807	169,579
Reduction (%)		−8,165 (−4.6)	−40,103 (−22.6)		−6,228 (−3.5)
Influenza deaths	448	431	361	444	431
Reduction (%)		−17 (−3.8)	−87 (−19.4)		−13 (−2.9)
Hospitalizations	1,719	1,650	1,377	1,705	1,653
Reduction (%)		−69 (−4.0)	−342 (−19.9)		−52 (−3.0)
Complications	18,121	17,213	13,786	17,984	17,276
Reduction (%)		−908 (−5.0)	−4,335 (−23.9)		−708 (−3.9)
QALYs lost	4,207.06	4,041.64	3,360.03	4,174.10	4,045.68
QALYs gained		+165.42	+847.03		+128.42
**Payer perspective (€, reimbursable medical/non-medical)**					
Vaccination costs	6,768,929	7,231,165	8,356,526	6,768,966	7,014,876
Influenza costs	8,364,143	7,902,975	6,279,774	8,302,969	7,973,380
*Total Payer costs*	*15,133,072*	*15,134,140*	*14,636,299*	*15,071,935*	*14,988,256*
Incremental cost		+1,068	−496,773		−83,679
**Cost/QALY gained**		**6.46** **<50k threshold**	**DOMINANT**		**DOMINANT**
**Societal perspective (€, Payer + non-reimbursable medical/non-medical and indirect)**					
Payer perspective	15,133,072	15,134,140	14,636,299	15,071,935	14,988,256
Vaccination costs	2,837,291	3,031,043	3,502,754	2,837,306	2,940,383
Influenza costs	1,445,977	1,379,083	1,120,291	1,435,440	1,384,519
Indirect costs	75,317,225	71,650,752	57,767,239	74,779,383	72,057,785
*Total Societal costs*	*94,733,565*	*91,195,019*	*77,026,584*	*94,124,064*	*91,370,943*
Incremental cost		−3,538,546	−17,706,981		−2,753,121
**Cost/QALY gained**		**DOMINANT**	**DOMINANT**		**DOMINANT**

^a^The first comparisons (Base case versus Strategy A and B) used the same random number sequence; while the second comparison (Base case versus Strategy C) used another random number sequence. As different random number sequences were used in the first and second comparison, the corresponding base case results differed slightly, reflecting the stochastic nature of the simulations (all results are arithmetic means of 1,000 simulations). Abbreviations: HR, high-risk; m, months; QALY, quality-adjusted life-year; y, years; €: Euro.

### Scenario analyses

As expected, reducing the health benefit discount rate from 3% to 1% in Scenario analysis 1 resulted in more QALYs gained with each strategy versus the base case. Strategy A became more cost-effective (€5/QALY gained, payer perspective) and remained dominant from a societal perspective, while Strategies B and C remained dominant from both payer and societal perspectives (Table 3).

**Table 3.** Scenario analyses results.

	**All children**				**High-risk children**	
	**Base case** **for A and B**^a^	**Strategy A (40% uptake, 6m-4y)**	**Strategy B (40% uptake, 6m-17y)**	**Base case for C^a^**		**Strategy C (90% uptake, HR 6m-17y)**
**Scenario 1: QALYs discounted at 1%, costs at 3%**						
QALYs lost	5,613.34	5,401.05	4,505.19	5,567.86		5,399.02
QALY gain		+212.29	+1,108.15			+168.84
Total Payer costs	15,133,072	15,134,140	14,636,299	15,071,935		14,988,256
Incremental cost		+1,068	−496,773			−83,679
**Cost/QALY**		**5.03** **<€50k threshold**	**DOMINANT**			**DOMINANT**
Total Societal costs	94,733,565	91,195,019	77,026,584	94,124,064		91,370,943
Incremental cost		−3,538,546	−17,706,981			−2,753,121
**Cost/QALY**		**DOMINANT**	**DOMINANT**			**DOMINANT**
**Scenario 2: German baseline utility (no differentiation by risk status)**						
QALYs lost	4,312.86	4,142.46	3,442.61	4,279.15		4,147.23
QALY gain		+170.40	+870.25			+131.92
Total Payer costs	15,133,072	15,134,140	14,636,299	15,071,935		14,988,256
Incremental cost		+1,068	−496,773			−83,679
**Cost/QALY**		**6.27** **< €50k threshold**	**DOMINANT**			**DOMINANT**
Total Societal costs	94,733,565	91,195,019	77,026,584	94,124,064		91,370,943
Incremental cost		−3,538,546	−17,706,981			−2,753,121
**Cost/QALY**		**DOMINANT**	**DOMINANT**			**DOMINANT**

^a^See explanations in Table 2. Abbreviations: HR, high-risk; m, months; QALY, quality-adjusted life-year; y, years; €: Euro.

In Scenario analysis 2, using German baseline utility data from the general population (with no distinction by healthy/high-risk status) slightly increased the QALY gains in each strategy and did not change the overall results (Table 3). Although high-risk groups are more adversely affected by influenza, they made up a small proportion of the total population in the model (i.e., 3–6% of children, 14.2% aged 16–59 years, and 47.1% aged 60+ years).^18^

### Probabilistic sensitivity analysis

**Figure 2.**
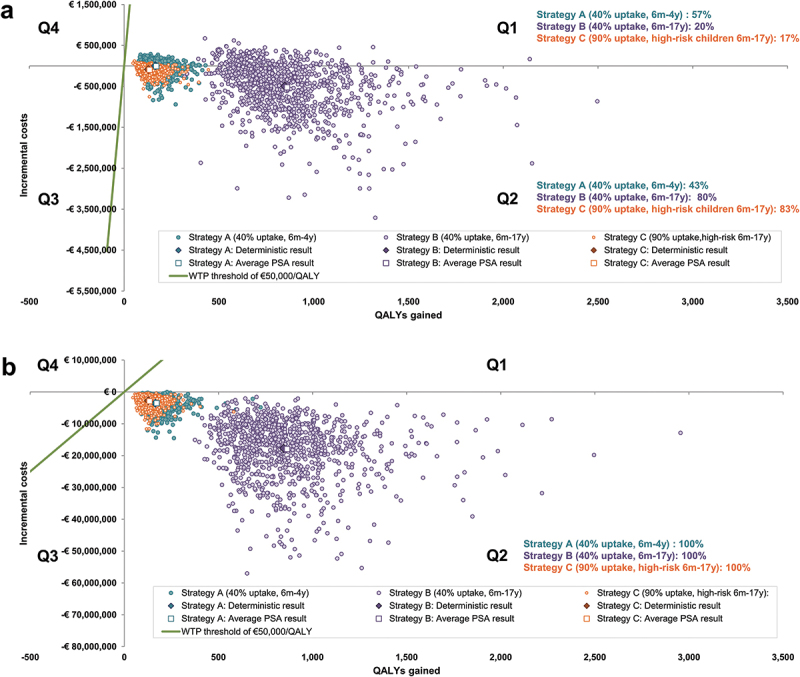
Cost-Effectiveness plane (1,000 PSA runs per strategy) for a German population of 100,000 individuals over 20 years (a) Payer perspective, (b) Societal perspective.

Abbreviations: m, months; PSA, probabilistic sensitivity analysis; Q, quadrant; QALY, quality-adjusted life-year; WTP, willingness-to-pay; y, years.

When considering uncertainty in both economic and epidemiologic parameters, Figure 2 shows that most points on the cost-effectiveness plane are in quadrant (Q) 2 indicating a health gain at a cost-saving. From the payer and societal perspectives, the probability that Strategies A, B and C are cost-effective at a threshold of €50,000/QALY gained was 100%. From the payer perspective, all PSA cost-effectiveness results remained below €3,170/QALY gained, with 57%, 20% and 17% of Strategy A, B and C results, respectively, in Q1 (indicating health gains at an increased cost versus the base case) and the rest in Q2. From a societal perspective, all three strategies produced health gains at a cost-saving versus the base case.

## Discussion

As children play a key role in the transmission of influenza,^5,48^ increasing QIV uptake in children significantly reduced the number of cases, complications and associated deaths in all age groups of the population, thereby reducing influenza management costs and productivity losses for the healthcare system and society. The model predicted that the strategy of increasing vaccine uptake to 40% in all children, healthy and high-risk, aged 6 months to 17 years would result in the largest health benefits and cost savings. Each year in Germany, this strategy would prevent on average 1.66 million influenza cases (more than 179,000 complications and 14,000 hospitalizations) and 3,600 influenza deaths, with an annual cost-saving of around €20.5 million (payer perspective) or €731.3 million (societal perspective). Increasing uptake to 40% in all young children (6 months to 4 years) was the next best strategy in terms of increasing population health gains (preventing on average 337,215 influenza cases per year), with cost-savings from prevention of influenza nearly compensating for the increased vaccination costs. This strategy, therefore, had comparable costs to the current situation but significantly greater benefits, resulting in a highly cost-effective incremental €6 per QALY gained. The final strategy modeled, increasing uptake to 90% only in high-risk children aged 6 months to 17 years, provided substantial cost savings (€3.5 million and €113.7 million from the payer and societal perspective, respectively) but fewer health gains (preventing on average 257,216 annual influenza cases) compared with strategies in both healthy and high-risk children.

Two other German economic analyses, also based on dynamic models, explored the impact on the entire population of expanding influenza vaccination to healthy children. The first one, conducted in 2015, assessed the impact of childhood vaccination with intranasal trivalent live-attenuated influenza vaccine (LAIV),^49^ and the second, by the Robert Koch Institute in 2020, assessed the impact of vaccinating with QIV, as QIV is currently recommended over TIV in Germany since the 2018/2019 influenza season.^41^ The first economic analysis, assuming a 50% LAIV uptake rate (versus 40% in our analysis) in children aged 2–17 years (versus from 6 months old in our analysis), estimated 16 million influenza cases of all ages would be prevented over 10 years, resulting in a highly cost-effective incremental cost-effectiveness ratio (ICER) of €1,228 (payer perspective) or cost-savings of €3.4 billion (societal perspective).^49^ While this analysis presents comparable case reductions to our analysis, the estimated cost-savings were lower, possibly due to the inclusion of only two complications of influenza (AOM and CAP), whereas our analysis also considered other relatively frequent complications such as bronchitis and URTI. In addition, the vaccine efficacy and cost per dose of LAIV (efficacy 80% and cost €20.20) in this study were considerably higher than for QIV in our analysis (efficacy range 50–69% for age group 2–17 years and cost of €13.11). The second economic study, assuming a QIV uptake rate of 40% in children, also only included two possible complications (AOM and/or CAP) with rates estimated from a German claims database analysis.^41^ The overall rates of these complications and of hospitalizations were fairly comparable with our analysis, however the most common complication (URTI) included in our analysis was not included, resulting in, for example, different hospitalization costs. Despite these differences, this analysis also concluded that vaccinating all children was highly cost-effective (ICER of €998) from a payer perspective and cost-saving from a societal perspective, even in a scenario analysis assuming no herd immunity and no subsequent influenza prevention in older age groups (ICER of €12,560). This analysis also found that expanding vaccination to a larger age group (in this case, all children aged 2–17 years) provided more health gains and remained cost-effective (i.e., ICER of €1,706).^41^

European countries are committed to reducing the burden of influenza, as recommended by the WHO.^50,51^ A systematic review published in 2017 found that switching from TIV to QIV provided both public health and economic benefits.^52^ With a growing population of older adults in Europe, who are at higher risk of morbidity and mortality from influenza, childhood vaccination can play a key role in achieving this target, as supported by increasing evidence from other countries. A systematic review of influenza immunization cost-effectiveness analyses was published in 2017,^53^ including high-quality studies in Europe that compared vaccinating all children versus high-risk children only. In Italy, in a cost-utility analysis in 2007, vaccinating all children aged 6 months to 2 years, or aged 6 months to 5 years, was cost-effective from a payer perspective (i.e., ICER of €13,333 and €10,000, respectively) compared to vaccinating only high-risk children. A cost-benefit analysis in Finland in 2006 found that vaccinating all children aged 6 months to 13 years was cost-saving and the dominant strategy compared to vaccination of high-risk children only. Similarly, in a cost-utility analysis in England and Wales in 2012, highly cost-effective ICERs were predicted with a universal vaccination policy for all children (using TIV in various age groups) versus a targeted high-risk children vaccination policy (i.e., ICERs of £192 for vaccination of children aged 2–4 years, £403 for children aged 2–10 years, and £429 for children aged 2–18 years, from a payer perspective). This study included the benefits of herd immunity in a dynamic transmission model.

Routine influenza vaccination of all children aged 2 and 3 years was introduced in England in the 2013/2014 influenza season. Based on published and real-world data, a model to assess the impact of this policy predicted a reduction of around 6–11% across the whole population in influenza-related GP visits, respiratory hospitalizations, and deaths.^54^ Annual vaccination increased each year and from the 2020/2021 influenza season, annual vaccination is recommended for all children aged 2 to 11 years.^55^

In France, the impact of extending influenza vaccination to all children aged 2–17 years (with an uptake of 50%) was modeled over 10 years, considering the effects on the entire population from herd immunity.^56^ The model estimated that each year, vaccination would directly prevent 865,000 cases among children under 18 years (approximately 60% reduction), and indirectly prevent 1.2 million cases in adults over 18 years (approximately 30% reduction), as well as preventing 613 deaths. From a payer perspective, this led to a cost-effective ICER of €18,001/life-year gained. In line with the findings of our analysis, this study also reported less cost-effective outcomes when vaccinating fewer children i.e., only children aged 2–6 years, as the indirect impact of vaccination is reduced when less children are vaccinated.^56^

Implementation of childhood influenza vaccination in Germany could be achieved through the regular medical contacts that occur for children under 4 years, where their vaccination status is also controlled. For older school-age children, real-world studies in high- and middle-income countries have shown that school-based programs can be beneficial settings to reach a large number of children in a short time.^57–59^

Our analysis had several limitations due to gaps in German input data for the model, which required the use of assumptions (based on Advisory Board consultations), or data from other countries in the EEA. For example, a 10-times higher all-cause mortality rate for high-risk versus healthy cases was based on data from the United Kingdom,^60^ and disutility data were based on Spanish data^23^ in order to have granularity by age group and risk status. The same probabilities were applied to cases seeking or not seeking medical advice (i.e., using the same hospitalization and death rate from complications). For complications, outpatient treatment duration was based on hospital treatment duration. These parameter uncertainties were tested in the PSA. The model assumed overall vaccination uptake would be achieved over 5 years and would be twice as high in high-risk versus healthy children. The benefits of vaccination may be underestimated as, taking a conservative approach, the costs and health impact from chronic conditions and rehabilitation that can result from influenza infection were not modeled; cases could only develop one complication, and influenza death only occurred in hospitalized complications. In our analysis, effective NI treatment was assumed not to affect the risk of influenza-related complications; exploratory analyses showed a negligible impact of reducing the risk of complications following effective NI treatment (see Supplementary file: Exploratory analysis). Similar to some other works,^41,61,62^ the impact of vaccine adverse effects was not considered in the analysis. Although serious adverse events can occur in rare instances, the vast majority of reported adverse events are generally transient mild-to-moderate injection site or systemic effects, with a negligible cost and minimal impact on quality of life,^61^ thus unlikely to relevantly change the model results and overall study conclusion.

The ongoing coronavirus disease 2019 (COVID-19) pandemic highlights the particular vulnerability of older adults and other risk groups in society from respiratory infections. Vaccination services have been disrupted during lockdown measures in Germany, although vaccination remains an essential tool for public health protection across all age groups and to reduce the pressure on over-burdened healthcare systems. Thus, vaccination uptake against all vaccine-preventable diseases needs to be increased. Recent press releases have shown that as a result of the COVID-19 pandemic, there is increased awareness of the impact of infectious diseases resulting in an increase in influenza vaccine acceptance and demand in most EU countries in the 2020/21 season.^63^

In conclusion, a vaccination policy targeting all children, healthy and at high-risk, against influenza is a highly cost-effective (and cost-saving to society) measure for preventing disease in all age groups, including those at higher risk of morbidity and mortality. This analysis provides decision-makers with further evidence that strongly supports expanding influenza vaccination coverage to all children in Germany, because of the protection this offers to vaccinated children as well as the indirect protection for adults and older adults, through reduced transmission.

Plain Language Summary presents a summary of the context, outcomes, and impact of this study for healthcare providers.

## Supplementary Material

Supplemental Material
