# Supplementary material for: Cost-utility analysis of increasing uptake of universal seasonal quadrivalent influenza vaccine (QIV) in children aged 6 months and older in Germany
Source: Hum Vaccin Immunother. 2022 Apr 29;18(5):2058304. doi: 10.1080/21645515.2022.2058304 (PMC9248945; doi:10.1080/21645515.2022.2058304)
Supplement: Supplemental Material [file KHVI_A_2058304_SM0367.docx]

**Supplemental online materials**

**Manuscript title:** Cost-utility analysis of increasing uptake of universal seasonal quadrivalent influenza vaccine (QIV) in children aged 6 months and older in Germany

**Authors:** Daniel Molnar^a^, Anastassia Anastassopoulou^b^, Barbara Poulsen Nautrup^c^, Ruprecht Schmidt-Ott^a^, Martin Eichner^d,e^, Markus Schwehm^f^, Gael Dos Santos^a^, Bernhard Ultsch^b^, Rafik Bekkat-Berkani^g^, Alfred von Krempelhuber^b^, Ilse Van Vlaenderen^h^, Laure-Anne Van Bellinghen^h^

**Affiliations**

^a^GSK, Wavre, Belgium

^b^GSK, Munich, Germany

^c^EAH-Consulting, Aachen, Germany

^d^Epimos GmbH, Bischofsheim, Germany

^e^University of Tübingen, Germany

^f^ExploSYS GmbH, Leinfelden-Echterdingen, Germany

^g^GSK, Rockville, MD, USA

^h^CHESS in Health, Bonheiden, Belgium

Present affiliation for Ruprecht Schmidt-Ott: Regierung von Oberbayern, Munich, Germany.

**Corresponding author**

Daniel Molnar

GSK, Building W23 E1, Avenue Fleming 20, 1300 Wavre, Belgium

Phone No: +32 10 85 5111

Email: daniel.h.molnar@gsk.com

**Journal:** Human Vaccines & Immunotherapeutics

**Supplementary Table 1.** Model inputs: probabilities, (dis)utilities and costs for symptomatic cases.

| **Probability** | **Healthy** | **High-risk** | **Source / comments** |
| --- | --- | --- | --- |
| Influenza infection is symptomatic | 66.9% | 66.9% | • Carrat et al.^1^ |
| Seeking medical advice (i.e. GP or AE visit) | 0–4y: 20.23%  5–49y: 28.58%  50–64y: 37.48%  65-100y: 16.39% | All ages: 37.48% | • Dolk et al.^2^ who estimated the probabilities based on Ryan et al.^3^ and Tappenden et al.^4^ |
| GP visit (as opposed to AE visit) of those seeking medical advice | 0–4y: 77.81%  5–17y: 79.96%  18–49y: 87.75%  50-100y: 90.05% | 0–4y: 77.81%  5–17y: 80.38%  18–49y: 87.75%  50+y: 90.05% | • Derived from Simonsen et al.^5^ |
| Receiving NI in those seeking medical advice | 0–5m: 6.29%  6m–4y: 7.27%  5–17y: 11.00%  18-100y: 15.00% | 0–5m: 12.44%  6m–4y: 14.84%  5–17y: 17.00%  18–49y: 17.60%  50+y: 20.00% | • Derived from Aballea et al.^6^ and Reuss et al.^7^  • Probability of virus resistance to NI treatment was set to zero (RKI report^8^). |
| Influenza duration with or without effective NI | No NI: 7.5 days  NI: 6.5 days | No NI: 7.5 days  NI: 6.5 days | • Garcia et al.^9^ and Braun et al.^10^ |
| Any complication: with or without previously seeking medical advice (see Suppl. Table 2 for complications) | 0–4y: 14.05%  5–17y: 12.50%  18–49y: 7.61%  50–64y: 7.95%  65-100y: 10.34% | 0–4y: 18.29%  5–17y: 15.95%  18–49y: 12.32%  50–64y: 12.59%  65+y: 13.76% | • Derived from Meier et al.^11^  • The same probability of complication is assumed in patients with or without NI treatment.^10^ |
| Hospitalization for any complication | 0–5m: 3.54%  6m–4y: 2.27%  5–17y: 1.61%  18–49y: 1.83%  50–64y: 10.83%  65-100y: 36.24% | 0–5m: 2.72%  6m–4y: 1.76%  5–17y: 1.26%  18–49y: 4.71%  50–64y: 18.93%  65+y: 27.23% | • Estimated by combining complication rates from table below with influenza hospitalization rates in total population (using rate per 100,000 and attack rate from Uhart et al.^12^). The model assumed total population hospitalization rate applies to rate of hospitalization due to a complication |
| Influenza death after hospitalization for any complication | 0–5m: 0.00%  6m–4y: 0.50%  5–17y: 0.75%  18–49y: 0.00%  50–64y: 0.00%  65-100y: 38.02% | 0–5m: 0.00%  6m–4y: 0.50%  5–17y: 0.75%  18–49y: 9.57%  50–64y: 12.00%  65+y: 38.02% | • Estimated by combining probability of complication and hospitalization with death rate (using rate per 100,000 and attack rate from Uhart et al.^12^). The model assumed death only occurs in hospitalized patients as supported by Rothberg et al.^13,14^ |
| **(Dis)utilities** | **Healthy** | **High-risk** | **Source / comments** |
| Baseline utility | 0–17y: 0.99  18–49y: 0.97  50–64y: 0.96  65–84y: 0.93  85–100y: 0.73 | 0–17y: 0.96  18–49y: 0.94  50–64y: 0.87  65–84y: 0.80  85–100y: 0.54 | • No German utilities by age and risk status identified. Therefore, Spanish population values used, estimated from the EQ-5D by Garcia et al.^9^ |
| Disutility influenza no complication, or with outpatient complication | 0–17y: 0.41  18–49y: 0.47  50–64y: 0.36  65**-**100y: 0.32 | | • Garcia et al.^9^ |
| Disutility influenza with hospitalized complication | 0–17y: 0.54  18–49y: 0.60  50–64y: 0.58  65-100y: 0.56 | | • Garcia et al.^9^ |
| Scenario analysis: German baseline utility | 0–17y: 0.972  18–49y: 0.965  50–64y: 0.931  65–84y: 0.867  85–+y: 0.839 | | • Baseline utilities from Janssen and Szende^15^ (no difference between healthy and high-risk) |
| **Costs** | **Reimbursable (€)** | **Non-reimbursable (€)** | **Source / comments** |
| QIV +  administration costs | 13.11 + 7.70 |  | • Lauer Taxe^16^ mean (published official) prices weighted by market share  • Administration cost estimated from GOP 89111 and 89112, vaccine assumed to be administered by physician’s clinic, therefore no non-reimbursed costs |
| GP visits | 0–5m: 40.01  6m–4y: 38.06  5–17y: 30.96  18–49y: 28.01  50–64y: 30.56  65–84y: 34.20  85-100y: 37.27 |  | • Derived from the official medical fee schedule (EBM 2017^17^) using GOP 03000 or GOP 04000 and GOP 03040 |
| AE visits | 0–5m: 48.43  6m–4y: 46.48  5–17y: 39.38  18–49y: 36.43  50–64y: 38.98  65–84y: 42.62  85-100y: 45.69 |  | • Derived from the official medical fee schedule (EBM 2017^17^) |
| Other medication | Antibiotic:  2.20–2.31  NI: 24.27–42.28 | OTC medication: 5.22  NI: 5.00 (for 18-100+y)  Antibiotic: 0.95 (for 18-100y) | • Antibiotic costs were multiplied by the proportion of patients that actually received antibiotics according to Ehlken et al.^18^  • NI and antibiotic costs varied by age group  • NI costs: Reuss et al.^7^ (who assumed that the only NI treatment was oseltamivir), and official tariff list “Rote Liste 2017”,^19^ considering rebates for 2017  • Antibiotics costs: Ehlken et al.^18^ (who modeled the number and types of antibiotics prescribed in adults and children to treat influenza), and official tariff list “Rote Liste 2017”.^19^  • OTC: Lugnér et al.^20^ |
| Child sickness benefit (*Kinderkrankengeld*) | 0–4y: 67.85  5–17y: 48.56 |  | • Derived from Dolk et al.^2^ (costs adapted to 2017 price level)  • Non-medical reimbursed cost for child treated with influenza in hospital or as outpatient |
| Transportation |  | For vaccination, GP/AE or outpatient: 8.72  For hospital: 11.03 | • Derived from Wolleswinkel-van den Bosch et al.^21^ and Ehlken et al.^22^ |
| **Indirect costs (€)** | | | **Source / comments** |
| Absenteeism and presenteeism | 0–4y: 541.12  5–17y: 560.41  18–64y: 608.97 (influenza no complication or outpatient complication)  18–64y: 1,660.82 (influenza hospital with complication) | | • Mean labor costs estimated from “*Statistisches Jahrbuch 2017*” (statistical yearbook 2017).^23^  • Absenteeism and presenteeism (number of days) were obtained from Ehlken et al.^18^ Absenteeism and presenteeism costs in children correspond to the time adults must take off to care for their sick child (corrected for children sickness benefit) |
| Premature mortality productivity loss | 15–19y: 714  20–24y: 2,984  25–29y: 5,889  30–34y: 7,451  35–39y: 7,974  40–44y: 8,439  45–49y: 8,917  50–54y: 8,574  55–59y: 7,790  60–64y: 4,886  65–69y: 2,191  70–100y: 0 | | • Calculated for 2017, following the same approach as used by Dolk et al.^2^ Values were pooled for both genders, for healthy and high-risk individuals, and the average individual / general population adjusted for work participation rate |

Abbreviations: AE, Accident & Emergency; EBM, einheitlicher bewertungsmaßstab; GOP, gebührenordnungsposition (Fee schedule positions); GP, general practitioner; m, month(s); NI, neuraminidase inhibitor; OTC, over-the-counter; RKI, Robert Koch Institute; QIV, quadrivalent influenza vaccine; y, year(s); €: Euro.

**Supplementary Table 2.** Proportion of specific complications (in those with any complication), complication costs and duration of treatment.

|  | **Proportion of complication in Healthy**  **/High-risk** | | | | | **Hospital cost (€)** | **Outpatient cost (€)** | **Duration of Tx (days)** | |
| --- | --- | --- | --- | --- | --- | --- | --- | --- | --- |
|  | **0–4y** | **5–17y** | **18–49y** | **50–64y** | **65-100y** | **R**  **/Non-R ≥18y** | **R<18y**  **/R≥18y**  **/Non-R ≥18y** | |  |
| Bronchitis | 4.85% | 7.17% | 14.51% | 24.98% | 28.47% | 3,023.35 | 63.88 | | 6.59 |
|  | 4.31% | 9.98% | 18.76% | 29.87% | 30.01% | 65.86 | 58.88 | |  |
|  |  |  |  |  |  |  | 5.00 | |  |
| Pneumonia | 1.24% | 1.81% | 3.59% | 4.20% | 11.05% | 4,228.25 | 113.13 | | 8.71 |
|  | 1.85% | 2.39% | 3.23% | 4.83% | 11.37% | 86.78 | 108.13 | |  |
|  |  |  |  |  |  |  | 5.00 | |  |
| URTI | 63.04% | 64.22% | 67.95% | 58.37% | 47.65% | 2,243.09 | 71.87 | | 5.61 |
|  | 62.01% | 62.48% | 63.22% | 53.67% | 40.56% | 56.09 | 66.87 | |  |
|  |  |  |  |  |  |  | 5.00 | |  |
| Cardiac | 0.00% | 0.02% | 0.08% | 0.57% | 0.94% | 3,148.16 | 393.93 | | 6.23 |
|  | 0.00% | 0.33% | 0.83% | 3.58% | 6.92% | 62.29 | 388.93 | |  |
|  |  |  |  |  |  |  | 5.00 | |  |
| Renal | 0.09% | 0.09% | 0.10% | 0.32% | 0.52% | 4,541.04 | 393.93 | | 9.62 |
|  | 0.00% | 0.11% | 0.28% | 0.36% | 1.41% | 96.22 | 388.93 | |  |
|  |  |  |  |  |  |  | 5.00 | |  |
| CNS | 0.73% | 0.95% | 1.65% | 1.29% | 2.19% | 3,421.28 | 393.93 | | 6.67 |
|  | 0.00% | 0.36% | 0.92% | 0.89% | 2.70% | 66.71 | 388.93 | |  |
|  |  |  |  |  |  |  | 5.00 | |  |
| AOM | 29.33% | 24.40% | 8.81% | 3.72% | 2.19% | 1,475.35 | 65.81 | | 3.22 |
|  | 31.42% | 22.50% | 8.69% | 2.86% | 1.29% | 32.24 | 60.81 | |  |
|  |  |  |  |  |  |  | 5.00 | |  |
| GI bleeding | 0.73% | 1.35% | 3.32% | 6.55% | 6.99% | 1,889.36 | 393.93 | | 4.23 |
|  | 0.41% | 1.84% | 4.07% | 3.94% | 5.74% | 42.27 | 388.93 | |  |
|  |  |  |  |  |  |  | 5.00 | |  |
| **Source and comments:** | | | | | | | | | |
| Proportions | Derived from Meier et al.^11^ Complications assumed to be mutually exclusive. | | | | | | | | |
| Hospital cost | For each complication, DRGs and number of cases were defined from G-DRG-Browser 2017.^24^ For each DRG, relative weight and mean length of stay were derived from “*Fallpauschalenkatalog*” for 2017.^25^ Mean weighted costs (by number of cases) were calculated considering federal base rate for 2017 (€3,376.11).^26^ Mean length of stay were calculated, weighted by number of cases per DRG.  Non-reimbursable costs: €10 co-payment per hospital day for patients ≥18y (maximum €280).^27^ | | | | | | | | |
| Outpatient cost | Outpatient complication treatment costs were derived from Dolk et al.^2^ and based on the following estimates: bronchitis, pneumonia and URTI were derived from Ehlken et al.,^22^ and GI bleeding was approximated from Chevat et al.^28^ Due to missing data, cardiac, renal, and CNS complication costs were assumed similar to GI bleeding. Finally, physician fees for AOM were derived from Wolleswinkel-van den Bosch et al.,^21^ with antibiotic costs in 60.8% of cases.  In patients ≥18y, a co-payment of €5 was assumed, representing co-payment for prescribed drugs costing €5–50^27^. | | | | | | | | |
| Duration of Tx | Duration of hospitalization was estimated from mean length of stay as calculated from DRGs for each complication.^24^ Mean length of stay was derived from “*Fallpauschalenkatalog*” for 2017^25^ and weighted by number of cases per DRG. The model assumed the same duration for hospitalization and outpatient treatment per complication. | | | | | | | | |
| Non-R | Note: all non-reimbursed costs are for patients ≥18y. | | | | | | | | |

Abbreviations: AOM, acute otitis media; CNS, central nervous system; DRG, diagnosis-related group; G-DRG, German diagnosis-related group; GI, gastrointestinal; R, reimbursed; Tx, duration of hospital or outpatient treatment for a complication; URTI, upper respiratory tract infection; y, year(s); €, Euro.

**Note on demographic parameters in 4Flu and e4Flu:** Due to the demographic nature of the German population, it is estimated that the number of inhabitants will decline over the 20-year evaluation period (2017–2036). Although we applied this forecast in the 4Flu model, the published results extrapolated back to the original starting population of 100,000 individuals. The e4Flu model, however, reports results based on the non-extrapolated demographic forecast (i.e. starting population of 100,000 that declines to 94,789 by 2036).

**Supplementary exploratory analysis: Neuraminidase inhibitor treatment on reduced risk of complications**

Neuraminidase inhibitors (NI) may reduce the risk of influenza-related complications.^29^ In exploratory analyses, the impact of decreasing the probability of complications following effective NI treatment by 10 to 50% was modelled in all age and risk groups simultaneously. The analysis found that the cost savings would decrease by 0.07% to 0.41%, and the quality-adjusted life-year gains by 0.29% to 1.49%. Thus, these analyses showed a negligeable impact on the base case results.

**References**

1. Carrat F, Vergu E, Ferguson NM, Lemaitre M, Cauchemez S, Leach S, Valleron AJ. Time lines of infection and disease in human influenza: a review of volunteer challenge studies. Am J Epidemiol. 2008; 167(7):775-785. doi: 10.1093/aje/kwm375.

2. Dolk C, Eichner M, Welte R, Anastassopoulou A, Van Bellinghen LA, Poulsen Nautrup B, Van Vlaenderen I, Schmidt-Ott R, Schwehm M, Postma M. Cost-Utility of Quadrivalent Versus Trivalent Influenza Vaccine in Germany, Using an Individual-Based Dynamic Transmission Model. PharmacoEconomics. 2016; 34(12):1299-1308. doi: 10.1007/s40273-016-0443-7.

3. Ryan J, Zoellner Y, Gradl B, Palache B, Medema J. Establishing the health and economic impact of influenza vaccination within the European Union 25 countries. Vaccine. 2006; 24(47-48):6812-6822. doi: 10.1016/j.vaccine.2006.07.042.

4. Tappenden P, Jackson R, Cooper K, Rees A, Simpson E, Read R, Nicholson K. Amantadine, oseltamivir and zanamivir for the prophylaxis of influenza (including a review of existing guidance no. 67): a systematic review and economic evaluation. Health Technol Assess. 2009; 13(11):iii, ix-xii, 1-246. doi: 10.3310/hta13110.

5. Simonsen KA, Hunskaar S, Sandvik H, Rortveit G. Primary care utilization among patients with influenza during the 2009 pandemic. Does risk for severe influenza disease or prior contact with the general practitioner have any influence? Fam Pract. 2015; 32(1):56-61. doi: 10.1093/fampra/cmu072.

6. Aballea S, Chancellor J, Martin M, Wutzler P, Carrat F, Gasparini R, Toniolo-Neto J, Drummond M, Weinstein M. The cost-effectiveness of influenza vaccination for people aged 50 to 64 years: an international model. Value Health. 2007; 10(2):98-116. doi: 10.1111/j.1524-4733.2006.00157.x.

7. Reuss A, Dehnert M, Buda S, Haas W. Differential use of antivirals for treatment of patients with influenza A(H1N1)pdm09 in Germany. Influenza Other Respir Viruses. 2013; 7(6):1427-1432. doi: 10.1111/irv.12152.

8. Robert Koch Institute (RKI). Report on the epidemiology of influenza in Germany, season 2015/2016. 2016 [accessed 2020 Nov 4]. https://influenza.rki.de/SAISONBERICHTE/2015.PDF.

9. Garcia A, Ortiz de Lejarazu R, Reina J, Callejo D, Cuervo J, Morano Larragueta R. Cost-effectiveness analysis of quadrivalent influenza vaccine in Spain. Hum Vaccin Immunother. 2016; 12(9):2269-2277. doi: 10.1080/21645515.2016.1182275.

10. Braun S, Behrens T, Kulp W, Eberle A, Greiner W, Ahrens W, von der Schulenburg JMG. Neuraminidasehemmer in der Therapie und Postexpositionsprophylaxe der Influenza. 2005 [accessed 2021 Feb 24]. http://portal.dimdi.de/de/hta/hta_berichte/hta116_bericht_de.pdf.

11. Meier CR, Napalkov PN, Wegmuller Y, Jefferson T, Jick H. Population-based study on incidence, risk factors, clinical complications and drug utilisation associated with influenza in the United Kingdom. Eur J Clin Microbiol Infect Dis. 2000; 19(11):834-842. doi: 10.1007/s100960000376.

12. Uhart M, Bricout H, Clay E, Largeron N. Public health and economic impact of seasonal influenza vaccination with quadrivalent influenza vaccines compared to trivalent influenza vaccines in Europe. Hum Vaccin Immunother. 2016; 12(9):2259-2268. doi: 10.1080/21645515.2016.1180490.

13. Rothberg MB, Fisher D, Kelly B, Rose DN. Management of influenza symptoms in healthy children: cost-effectiveness of rapid testing and antiviral therapy. Arch Pediatr Adolesc Med. 2005; 159(11):1055-1062. doi: 10.1001/archpedi.159.11.1055.

14. Rothberg MB, Bellantonio S, Rose DN. Management of influenza in adults older than 65 years of age: cost-effectiveness of rapid testing and antiviral therapy. Ann Intern Med. 2003; 139(5 Pt1):321-329. doi: 10.7326/0003-4819-139-5_part_1-200309020-00007.

15. Janssen B, Szende A. Population norms for the EQ-5D. In: Szende A, Janssen B, Cabases J, eds. Self-reported population health: An international perspective based on EQ-5D. Dordrecht (NL): New York: Springer, 2014.

16. CGM Lauer. Lauer Taxe. [accessed 2020 Nov 4]. https://www.lauer-fischer.de/LF/Seiten/Verwaltung/Kundencenter/1.aspx.

17. Kassenarztliche Bundesvereinigung (KBV). Einheitlicher Bewertungsmaßstab (EBM) 2017. 2017 [accessed 2020 Nov 4]. http://www.kbv.de/html/ebm.php.

18. Ehlken B, Anastassopoulou A, Hain J, Schroder C, Wahle K. Cost for physician-diagnosed influenza and influenza-like illnesses on primary care level in Germany--results of a database analysis from May 2010 to April 2012. BMC Public Health. 2015; 15:578. doi: 10.1186/s12889-015-1885-0.

19. ROTE LISTE 2017 Buchausgabe Einzelausgabe. Frankfurt, Germany: Rote Liste Service GmbH, 2017.

20. Lugnér AK, van Boven M, de Vries R, Postma MJ, Wallinga J. Cost effectiveness of vaccination against pandemic influenza in European countries: mathematical modelling analysis. BMJ. 2012; 345:e4445. doi: 10.1136/bmj.e4445.

21. Wolleswinkel-van den Bosch JH, Stolk EA, Francois M, Gasparini R, Brosa M. The health care burden and societal impact of acute otitis media in seven European countries: results of an Internet survey. Vaccine. 2010; 28 Suppl 6:G39-52. doi: 10.1016/j.vaccine.2010.06.014.

22. Ehlken B, Ihorst G, Lippert B, Rohwedder A, Petersen G, Schumacher M, Forster J, Group PS. Economic impact of community-acquired and nosocomial lower respiratory tract infections in young children in Germany. Eur J Pediatr. 2005; 164(10):607-615. doi: 10.1007/s00431-005-1705-0.

23. Statistisches Bundesamt. Statistisches jahrbuch Deutschland und Internationales 2017. Germany, 2017.

24. Institut für das Entgeltsystem im Krankenhaus (InEK). G-DRG Browser 2017 [accessed 2020 Nov 4]. http://www.g-drg.de/Datenbrowser_und_Begleitforschung/G-DRG-Report-Browser/G-DRG-Report-Browser_2017.

25. Institute for Remuneration System in the Hospital (InEK). Fallpauschalenkatalog 2017. 2017 [accessed 2019 Feb 24]. https://www.g-drg.de/G-DRG-System_2017/Fallpauschalen-Katalog2/Fallpauschalen-Katalog_2017.

26. GKV-Spitzenverband. Bundesbasisfallwert (BBFW). [accessed 2017 Aug 21]. https://www.gkv-spitzenverband.de/krankenversicherung/krankenhaeuser/budgetverhandlungen/bundesbasisfallwert/bundesbasisfallwert.jsp.

27. Krankenkassen Deutschland. Gesetzlich vorgeschriebene Leistungen. [accessed 2021 Feb 24]. https://www.krankenkassen.de/gesetzliche-krankenkassen/leistungen-gesetzliche-krankenkassen/gesetzlich-vorgeschriebene-leistungen/.

28. Chevat C, Pena BM, Al MJ, Rutten FF. Healthcare resource utilisation and costs of treating NSAID-associated gastrointestinal toxicity. A multinational perspective. PharmacoEconomics. 2001; 19 Suppl 1:S17-32. doi: 10.2165/00019053-200119001-00002.

29. Paules C, Subbarao K. Influenza. Lancet. 2017; 390(10095):697-708. doi: https://doi.org/10.1016/s0140-6736(17)30129-0.
